# Design and evaluation of two educational media in the form of animation and games to promote the cutaneous leishmaniasis prevention behaviors in adolescent female

**DOI:** 10.1186/s12889-022-14772-8

**Published:** 2022-12-07

**Authors:** Masoumeh Alidosti, Hossein Shahnazi, Zahra Heidari, Fereshteh Zamani-Alavijeh

**Affiliations:** 1 PhD Student of Health Education and Promotion, Student Research Committee, School of Health, Isfahan University of Medical Sciences, Isfahan, Iran; 2 Associate Professor of Health Education and Promotion, Department of Health Education and Promotion, School of Health, Isfahan University of Medical Sciences, Hezar Jarib Avenue, Isfahan, 81676-36954 Iran; 3 Assistant Professor of Biostatistics, Department of Epidemiology and Biostatistics, School of Health, Isfahan University of Medical Sciences, Isfahan, Iran; 4 Professor of Health Education and Promotion, Department of Health Education and Promotion, School of Health, Isfahan University of Medical Sciences, Isfahan, Iran

**Keywords:** Animation, Games, Education, Intervention, Extended Parallel Process Model, Cutaneous leishmaniasis, Adolescents

## Abstract

**Background:**

Cutaneous leishmaniasis (CL) has social and psychological effects on different groups, especially adolescents and young girls in 98 countries of the world, in addition to the economic burden. Therefore, it is necessary to apply effective methods for CL prevention. In this study, educational messages were designed based on Extended Parallel Process Model in both forms of animation and game, whose effect on the cutaneous leishmaniasis prevention behaviors was evaluated in adolescent female students.

**Methods:**

This experimental study was carried out from January to September 2021 on 275 adolescent female students in Isfahan province, Iran. Cluster sampling method was used, and then the students were randomly divided into three groups, namely animation education, game education, and control groups. The educational intervention was performed with two new media in the form of animation and games. The data collected before and two months after the education through a valid and reliable researcher-made questionnaire were analyzed in SPSS24 software using statistical tests of ANOVA, Chi-square, paired t and analysis of covariance (ANCOVA).

**Results:**

The mean age of the participants was 14.07 ± 0.94. The mean scores of behavior in the animation group (60.60 ± 23.00), the game group (61.70 ± 22.05), and the control group (66.13 ± 24.62) were not significantly different prior to the education. However, after the education, there was a significant difference between the animation (80.66 ± 17.62) and game groups (82.58 ± 19.07) and the control group (69.79 ± 23.29) (*P* < 0.001). The mean scores of model constructs following the intervention (susceptibility, severity, response efficacy, and perceived self-efficacy) significantly increased in the animation and game groups compared to that in the control group (*P* < 0.05).

**Conclusion:**

The results showed that if educational programs contain a combination of threat and efficiency messages, CL-preventive behaviors in adolescents increase. Providing similar educational content with both game and animation methods indicated that both methods had an almost same effect. Although animation production is more costly, it has the advantage of being used in periods and for other adolescents.

**Supplementary Information:**

The online version contains supplementary material available at 10.1186/s12889-022-14772-8.

## Implications and contribution

Providing educational programs with a combination of threat and efficiency messages with game and animation methods is associated with an increase in cutaneous leishmaniasis (CL) prevention behaviors in adolescents.

## Introduction

Leishmaniasis is the second important disease transmitted by protozoan parasites after malaria, which is an important public health problem in 98 countries, including Iran [1, 2]. Isfahan is one of the endemic areas of this disease in Iran [3]. Although the cutaneous form of the disease is not usually fatal, it can lead to malignant or debilitating scars [4]. Difficult treatment, high medical costs, drug resistance, drug side effects, long-term wounds, the possibility of secondary infection, and unpleasant scar formation are some of the problems associated with this disease [5]. Therefore, it is necessary to prevent CL, but there are no safe and effective vaccines for this purpose [6] and educating people at risk to protect themselves from sandfly bites is believed to be one of the best methods [7].

### The necessity of CL prevention education in adolescent female students

The psychological effects of CL have been further reported in adolescents, particularly girls [8, 9]. The presence of scars on the face of women affects their marriage status more than men [10, 11]. On the other hand, students, as the future and human resources of any society, have a pivotal role in conveying health concepts [12]. Providing students with CL prevention education, they can act as a bridge to promote these behaviors in family and community [13]. Despite the educational interventions in this field, has not been used new educational methods such as showing animation, while animation can be a short way to achieve desirable changes in health [12, 14, 15]. Furthermore, game, which is a valuable educational tool [16] in adolescent health education, has a high potential [17]. In the present study, in addition to the above-mentioned factors, for the first time, the message design model was used to teach the preventive measures of cutaneous leishmaniasis.

### Selected theoretical framework

Health educators often motivate people to adopt healthy behaviors or change unhealthy ones by providing information in the form of health messages [18]. Theories of health-related behavior should be used in the design of health messages. These theories consider the perceived susceptibility of individuals and the perceived severity of a health concern as determinants in whether to employ protective measures or not. Another important concept in message design is the efficiency (self-efficacy and response efficacy). These concepts are expressed as important elements for the message in the Extended Parallel Process Model (EPPM) [19].

According to the EPPM, three reactions can be expected when both perceived threat and perceived efficacy are high; individuals are expected to accept the message and perform actions to avert the threat. This is called "*danger control*". When the perceived threat is high while the perceived efficacy is low, individuals may enter the "*fear control*" phase; they prevent fear by minimizing the message or denying the existence of danger rather than the threat. When perceived threat is low, people do "*not respond*" and may not even evaluate the effectiveness of the recommended action [20]. Consequently, those taking the threat more seriously, who are more confident about the ability to perform a behavior and its positive effects are more likely to perform protective behaviors and danger control [21]. The theoretical framework of the current study was based on EPPM.

According to the above-mentioned points, in the present study, educational messages based on Extended Parallel Process Model were designed in both forms of animation and game, whose effect on improving the cutaneous leishmaniasis prevention behaviors was evaluated in adolescent female students.

## Method

This experimental work was a three-group intervention whose results were assessed before and after it. The study was conducted from January to September 2021 on 275 high school female students in Isfahan province, Iran.

### Sample size and sampling method

With cluster sampling method among the endemic areas of Isfahan province (Iran), Borkhar area was selected. Out of the 16 governmental first girls’ high schools in Borkhar area, three were randomly selected. All their students were divided into one of the two intervention or control groups through a draw.

The number of groups was three (g = 3), assuming that the minimum detectable effect size is ∆ = 0.5, α = 0.05, and 1-β = 0.8. Based on the following sample volume formula, the required sample volume for each group was approximately equal to 90 students, being a total of 270 students and assuming a 0.5% drop of 285 [22].

$${n}_{1}=\left(1+\sqrt{g-1}\right)\frac{{({z}_{1-{\alpha}\!\left/ \!{2}\right.}+{z}_{1-\beta })}^{2}}{{\Delta }^{2}}+\frac{{z}_{1-{\alpha}\!\left/ \!{2}\right.}^{2}\sqrt{g-1}}{2(1+\sqrt{g-1})}=\left(1+\sqrt{4-1}\right)\frac{7.849}{{0.5}^{2}}+\frac{{1.96}^{2}\sqrt{4-1}}{2(1+\sqrt{4-1})}\cong 90$$

$$n={n}_{1}+(g-1){n}_{2}=90+(2*90)=270$$

**Fig.1:**
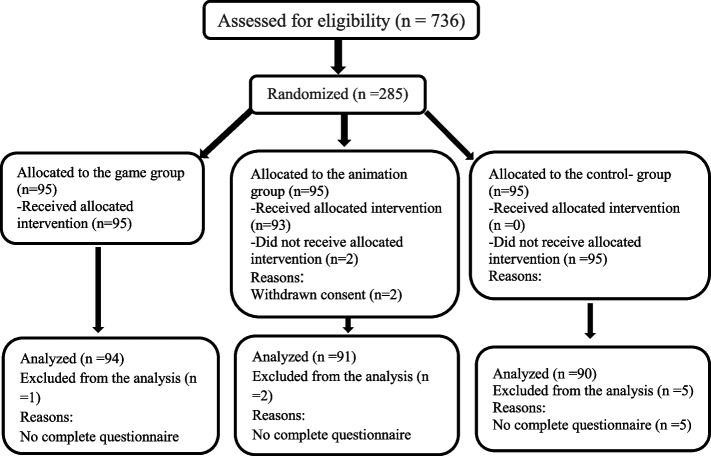
Flow diagram of the study participants

Out of the 736 students in the three selected schools, 285 were selected such that according to the sample size formula and with the probability of dropping samples, from each school, 95 students were simple randomly selected. The inclusion criteria were as follows: residence in the area for more than six months and student and parents' satisfaction to participate in the study. The exclusion criteria included immigration, not receiving education in the intervention groups for any reason, and unwillingness to continue cooperation at any stage of the study. Figure 1 shows the number of participants from the beginning to the end of the study (Fig. 1).

### Data collection tool and method

According to the literature review [6] and the opinions of the expert team, a questionnaire was prepared based on EPPM [23].

The questionnaire included seven demographic questions, in addition to 14 about knowledge, six about susceptibility, seven about severity, nine about self-efficacy, six about response efficacy, four about fear, 13 about fear control process, and 13 about danger control process (behavior and intention) [23].

To collect data, due to the prevalence of COVID-19 and school closures, a questionnaire was set in at https://survey.porsline.ir and for all the three groups of students, the relevant link was sent via telephone coordination, before and two months after the educational intervention. It took between 14 to 17 min for each student to complete the questionnaire. In the first part of the questionnaire, after introducing the researcher, explanations were given about the purpose of the research, how to participate in the study, confidentiality of the information, and optional participation in the research.

### Educational content

Based on the structures of EPPM, the educational content was organized using scientific sources, following which game cards and animation scenario were prepared. The evaluation team (four health education specialists and three healthcare workers) reviewed the messages, scenarios, characters, and spaces related to each of the media. The necessary corrections were made and the messages were provided with both game and animation methods.

A game that could be played by two to four students had cards with positive and negative scores. The negative score cards that were the sandfly attack card included threat messages, such as sandfly attack time and its hiding places (Table 1). The positive score cards had messages of efficiency (Table 1). During the game, once the attack card is exposed, the opponent must show a positive card and neutralize the attack and if the score of the positive card is lower than that of the attack, the next person must show another positive card to neutralize the attack and take the cards. In the end, the winner of the game is the one who has collected more positive cards.

**Table 1:**
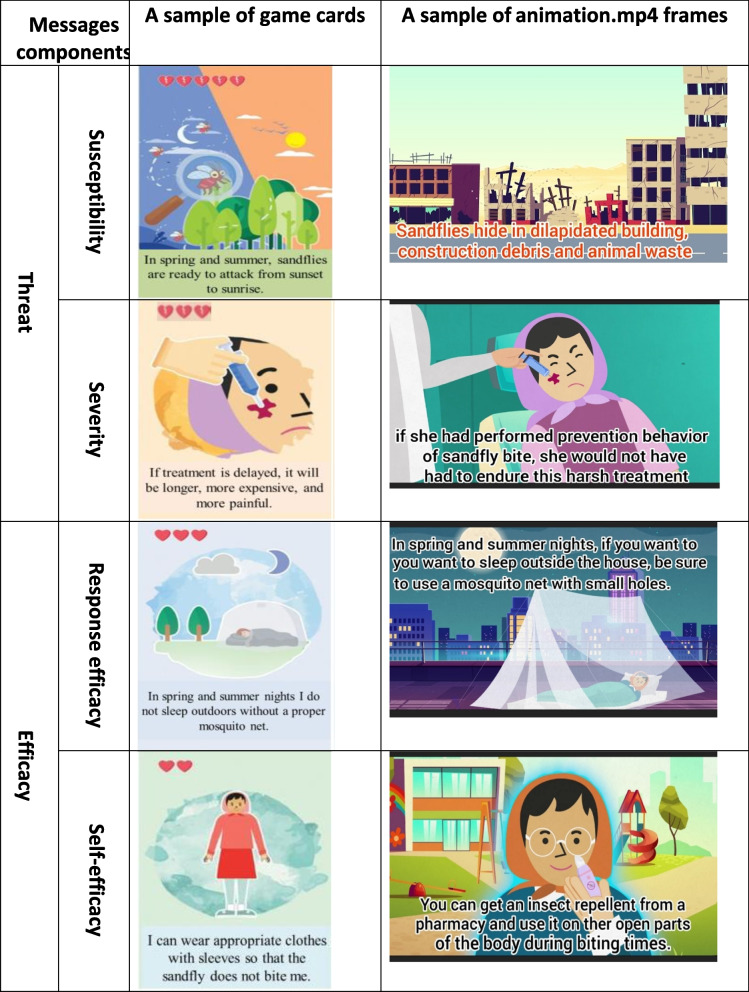
An example of game cards and animation frames based on elements of educational messages

Animation.mp4 tells the story of a girl suffering from CL sores on her face due to not having paid attention to the messages of threat and efficiency and now she has noticed the severity and hardness of the disease (Table 1). In the rest, the behaviors that she should have performed are demonstrated.

Threat messages focused on the sandfly bites time periods (in spring and summer from sunset to the sunrise of the next day), sandfly hideouts (sand dunes, dilapidated buildings, construction debris and garbage, animal wastes, cracks in the walls), painful wounds and difficult treatment, and scar development due to delay in medical treatment.

Efficiency messages focused on the ability to perform preventive behaviors. Effective behaviors are as follows: using appropriate clothing to cover all parts of the body, using insect repellents for open parts of the body, not going to ruined places, installing nets with small holes indoors as well as windows and air conditioner vents, using a suitable mosquito net for sleeping outdoors, taking actions to improve their environment to minimize sandfly threat (repairing cracks in the wall, not dumping garbage and construction debris around the house, and follow-up for their collection by the relevant organizations), and early treatment.

### Performing the intervention

Due to the prevalence of COVID-19, to prevent overcrowding, with the coordination of the school manager, telephone calls were made to the students of the two intervention schools, each of whom was asked to come with one of her parents at a certain time for receiving the educational package and learning how to use it.

In the game group, the game cards were distributed with their guide as well as explanations for the students and their parent. A WhatsApp Virtual Group was formed for the students, and to encourage them to send videos and photos since playing the card game, a competition was held, “What is the message of this card?”. Free internet code prize was awarded to six students via a draw.In the animation group, the students and their parents were provided with explanations along with the animation. Afterwards, a WhatsApp virtual group was formed for the students in this group. To ensure that they watch the animation and encourage them to pay attention to the scenes and messages, a competition was held, "What is the message of this scene from the animation?" and six students that answered correctly and completely were selected. Free internet code prize was awarded to them.

Appropriate feedback was given to all their answers in order to correct misconceptions and the education was done with questions and answers on WhatsApp.In the control group, no education was conducted by the research team during the study. Nevertheless, after completing the questionnaires in the second stage, to observe ethical principles, the animation was sent to these students.

### Data analysis

Data analysis was carried out with the help of SPSS version 24. The scores of all the sections were calculated to be between 0 to 100.. The score on the knowledge-related questions was as follows: correct answer "2", I do not know "1", and incorrect answer “0”. The perception- and fear control process-related questions, with a four-point Likert spectrum, ranged from Strongly agree “3” to Strongly disagree “0”. The questions about fear, with a five-point Likert spectrum, was between Very low “0” and Very high “4”. The danger control process-associated questions were on a four-point Likert spectrum (those about intention from Never “0” to From this month “3” and those about behavior from Rarely “0” to Always “3”). In the sections of knowledge, perceptions, and danger control process, higher scores indicated conditions that are more favorable whereas in the fear control process section, a lower score implied these conditions.

The study did not have missing data because the condition of answering all the questions was included in the online questionnaire so that they could complete and send it. Quantitative variables were reported as mean (standard deviation) while qualitative variables were expressed as numbers (percentage). The Kolmogorov–Smirnov test was applied to determine the normal distribution of the variables. Analysis of variance (ANOVA) and Chi-square test were employed to respectively compare the quantitative and qualitative variables among the three groups. To identify the within-group differences, paired-sample t-tests were performed. Analysis of covariance (ANCOVA) was carried out to compare the effect of the intervention on the three groups. A *P*-value of < 0.05 was considered to be statistically significant.

## Results

A total of 275 students participated throughout the study. The mean age of the participants in the animation group was 14.20** ± **1.06, which was 14.00** ± **0.96in the game group, and 14.02** ± **0.79 in the control group; ANOVA test showed that the mean age of the three groups was not significantly different (*P* = 0.30). In addition, Chi-square test illustrated that the demographic characteristics of the three groups were not of significant differences. About half of the students or their family members already had CL. More than two-thirds of them living in this area are non-native (Table 2).

**Table 2:** Comparison of the demographic data of the three groups

Variable		Animation group	Game group	control group	*P*- value
		(Number / Percentage)	(Number / Percentage)	(Number / Percentage)	
**Native**	**Native**	20(22%)	17(18.1%)	12(13.3%)	**0/31**
	**Non-native but resident**	71(78%)	77(81.9%)	78(86.7%)	
**Precedent CL in themselves or families**	**YES**	49(53.8%)	43(45.7%)	47(52.2%)	**0/50**
	**NO**	42(46.2%)	51(54.3%)	43(47.8%)	
**Type of housing**	**Old**	15(16.5%)	11(11.7%)	19(21.1%)	**0/22**
	**New**	76(83.5%)	83(83.3%)	71(78.9%)	
**Mother's education**	**Illiterate**	8(8.8%)	5(5.3%)	4(4.4%)	**0/07**
	**Elementary**	25(27.5%)	18(19.1%)	20(22.2%)	
	**Guidance**	19(20.9%)	16(17%)	17(18.9%)	
	**Diploma**	29(31.9%)	36(38.3%)	34(37.8%)	
	**University**	10(11%)	19(20.2%)	15(16.7%)	
**Father's education**	**Illiterate**	11(12.1%)	3(3.2%)	3(3.3%)	**0/21**
	**Elementary**	33(36.3%)	23(24.5%)	32(35.6%)	
	**Guidance**	21(23.1%)	21(22.3%)	25(27.8%)	
	**Diploma**	18(19.8%)	31(33%)	24(26.7%)	
	**University**	8(8.8%)	16(17%)	6(6.7%)	
**Grade**	**Seventh**	33(36.3%)	36(38.3%)	33(36.7%)	**0/87**
	**Eighth**	29(31.9%)	31(33%)	34(37.8%)	
	**Ninth**	29(31.9%)	27(28.7%)	23(25.6%)	

The results of paired t-test demonstrated a significant difference between the animation and game groups in terms of the mean scores variables studied two months after the education (*P* < 0.05). However, no significant differences were observed in the control group in any of the variables (Table 3). The results of ANOVA test showed that the mean scores of the evaluated variables in the three groups were not significantly different before the education, but were significantly different two months after that. According to the result of Tukey test, the difference was significant between the control group and the two intervention groups (*P* < 0.05) (Table 3). ANCOVA test revealed that the mean score change of variables in the two intervention groups was of significance. Accordingly, the mean score of fear and fear control reactions in the two intervention groups decreased significantly whereas the mean score of change of other variables increased significantly in these groups (*P* < 0.05) (Table 3).

**Table 3:** Comparison of the mean scores of the studied variables before and after the education in groups and between groups

Variable	Group	Before education	After education	Change	*P*-value*	*P*-value***
		**Mean ± SD**	**Mean ± SD**	**Mean ± SD**		
**Knowledge**	Animation *N* = 91	64.88** ± **14.10	80.33** ± **12.79	15.44** ± **13.99	*p* < 0.001	*p* < 0.001
	Game *N* = 94	65.00** ± **14.78	84.61** ± **14.68	19.60** ± **13.11	*p* < 0.001	
	Control *N* = 90	66.57** ± **15.98	65.14** ± **13.80	-1.42** ± **11.67	*p* = 0.25	
	*P*-value**	*p* = 0.70	*P* < 0.001	*p* < 0.001	-	
**Perceived susceptibility**	Animation *N* = 91	61.17 ± 14.75	75.80** ± **13.76	14.64 ± 10.61	*p* < 0.001	*p* < 0.001
	Game *N* = 94	64.65 ± 13.50	79.96** ± **12.86	15.30** ± **7.31	*p* < 0.001	
	Control *N* = 90	65.00 ± 15.34	66.97** ± **17.79	1.97** ± **13.12	*p* = 0.15	
	*P*-value**	*p* = 0.14	*P* < 0.001	*p* < 0.001	-	
**Perceived severity**	Animation *N* = 91	63.63 ± 20.10	77.65** ± **14.54	14.02 ± 10.46	*p* < 0.001	*p* < 0.001
	Game *N* = 94	67.32 ± 13.29	79.88** ± **14.76	12.56** ± **9.20	p < 0.001	
	Control *N* = 90	65.18 ± 16.85	66.61** ± **18.12	1.42** ± **10.99	*p* = 0.22	
	*P*-value**	*p* = 0.33	*P* < 0.001	*P* < 0.001	-	
**Perceived response efficacy**	Animation *N* = 91	66.78** ± **16.20	81.86** ± **18.36	15.07** ± **13.20	*p* < 0.001	*p* < 0.001
	Game *N* = 94	69.44** ± **12.84	86.34** ± **15.57	16.90** ± **13.03	*p* < 0.001	
	Control *N* = 90	70.30** ± **15.17	72.09** ± **17.20	1.79** ± **10.56	*p* = 0.11	
	*P*-value**	*p* = 0.24	*P* < 0.001	*P* < 0.001	-	
**Perceived self-efficacy**	Animation *N* = 91	71.30** ± **19.66	84.69** ± **14.28	13.39** ± **12.49	*p* < 0.001	*p* < 0.001
	Game *N* = 94	73.52** ± **14.50	87.78** ± **14.70	14.26** ± **12.04	*p* < 0.001	
	Control *N* = 90	76.00** ± **16.26	77.28** ± **18.46	1.27** ± **17.33	*p* = 0.48	
	*P*-value**	*p* = 0.17	*P* < 0.001	*P* < 0.001	-	
**Fear**	Animation *N* = 91	38.53** ± **23.61	31.59** ± **25.19	-6.93** ± **13.12	*p* < 0.001	*p* < 0.01
	Game *N* = 94	34.17** ± **18.25	28.39** ± **19.54	-5.78** ± **14.81	*p* < 0.001	
	Control N = 90	36.75** ± **32.21	38.54** ± **27.65	1.78** ± **29.62	*p* = 0.56	
	*P*-value**	*p* = 0.49	*P* < 0.05	*P* < 0.01	-	
**Fear control process**	Animation *N* = 91	40.20** ± **19.28	32.06** ± **19.71	-8.14** ± **7.15	*p* < 0.001	*p* < 0.001
	Game *N* = 94	39.99** ± **18.83	29.21** ± **19.25	-10.78** ± **7.65	p < 0.001	
	Control *N* = 90	40.81** ± **18.91	38.81** ± **17.59	-1.99** ± **16.45	*p* = 0.25	
	*P*-value**	*p* = 0.95	*P* < 0.01	*P* < 0.001	-	
**Danger control process**	Animation *N* = 91	63.77** ± **21.29	81.54** ± **19.29	17.77** ± **11.18	*p* < 0.001	*p* < 0.001
	Game *N* = 94	61.08** ± **20.13	83.31** ± **19.55	22.23** ± **11.95	*p* < 0.001	
	Control *N* = 90	66.35** ± **24.22	70.73** ± **25.81	4.37** ± **21.67	*p* = 0.059	
	*P*-value**	*p* = 0.26	*P* < 0.001	*P* < 0.001	-	

^*^P-value is based on Paired Sample t-Test

^**^P-value is based on Oneway ANOVA

^***^P-value is based on ANCOVA

## Discussion

Herein, for the first time, message design based on Extended Parallel Process Model (EPPM) in the form of animation and game was used to educate female students on CL risk prevention. Prior to the education, the mean score of danger control process ranged between 61 and 66 in the groups. These results shed light on the need to apply effective educational interventions. In this study, the use of animation and games respectively increased the mean score of CL danger control process by 17 and 22 points, and showed the effectiveness of this type of educational intervention in students.

Investigating the demographic data and studied variables before the intervention ensured the samples homogeneity in the three groups and the minimal effect of confounding variables on the study results. In the following, all the studied variables are discussed.

### Knowledge

Knowledge is a prerequisite for CL prevention educational programs [5]. The mean score of knowledge increased significantly after the educational intervention in the animation group and the game group. The results of other studies are in agreement with ours, indicating the effect of education on raising students’ knowledge about CL [24, 25]. In the study by Alharazi et al., although the participants were aware of CL, but they had poor overall knowledge about disease transmission, clinical presentation, treatment, and prevention. In addition, approximately half of the participants were able to differentiate sand flies from other mosquitoes [10]. These findings were similar to the results before the intervention in the present studyComparing the two groups of animation and game, the increase in knowledge was slightly higher in the game group compared with the animation group; although this difference was not significant, there is a growing belief in the potential of games and the experience of students and educators is positive using them [16]. The effectiveness of teaching through dynamic methods can also be deduced from the sayings of big figures; for example, the philosopher Confucius says:”Tell me and I will forget. Show me and I may remember. Involve me and I will understand” [16]. In the present work, it could be inferred that the education of health centers performed annually in endemic areas with traditional methods, such as lecturing in schools, may lead to "forgetting" while educating by showing animations is an example of "showing”, which results in “remembering", and education in game be an example of “involving” resulting in “activity". Of course, involvement in learning will be more effective; hence, in the study of Haruna et al., game method in students was an active and motivating method to find which has a greater affect [17].

### Perceived threats

Perceived threat is perceived susceptibility and perceived severity. It is of predictive factors for adopting preventive behaviors against CL [25]. Although no research has been conducted applying EPPM for education on cutaneous leishmaniasis prevention, perceived threat is from the structures of this model, which was investigated in the study by Ghodsi et al. [25] using the Health Belief model. In accordance with the present study, they showed the effectiveness of education in this structure.. In the current research, the educational media was different from those of previous papers, which can further attract students’ attention since novelty arouses the sense of curiosity and increases the desire for gaining knowledge [26].

### Perceived efficiency

Perceived efficiency includes response efficacy and self-efficacy [27]. About response efficacy, although a number of studies have considered it to be effective in CL-preventive recommendations [6, 28], no research was found in the field of CL providing education to improve the perceived response efficacy. While perceived response efficacy is an important mediator between self-efficacy and behavior, it is very important to convince the person that the recommended behaviors are effective [29]. The present study investigated this issue for the first time and showed the effectiveness of intervention in improving perceived response efficacy.

The present work, by showing prevention methods through game cards and animation, increased students' belief in their ability to apply the recommendations. Hashemi et al. used animation and game methods for improving self-efficacy, followed by which there was an increase in students' knowledge and preventive behaviors [14].

### Fear

Fear decreased significantly after the intervention in the animation and game groups. According to this model, very high levels of fear may lead to unhelpful and even destructive side effects [29]. Messages with a high level of fear will not be useful in promoting health behavior if they are not accompanied by some practical advice and will result in the fear control process [30]. Herein, an attempt was made to manage fear with messages, such as "Take preventive behaviors instead of being afraid of CL". Of course, the average fear score did not reach zero because there were messages of susceptibility and severity presented in the form of animation and game, causing the students to ascribe to themselves the experiences of others in the form of stories through curiosity and reinforcing guessing [31]. Therefore, a level of fear was maintained, which is necessary for starting and continuation of preventive behaviors [32]. It is not possible to compare the results since no study has investigated or intervened in this field.

### Fear control process

In the fear control process, there were three different responses, namely defensive avoidance of the message, message minimization, and perceived manipulation [33], each of which is a type of maladaptive reaction against risk massages. If the interventions focus only on perceived threat and do not pay attention to perceived efficiency, fear control process happens [21]. In this case, education simply fails with defensive avoidance reactions, such as "I do not want to talk and hear about CL." or message minimizations, such as "CL talk and images are false and exaggerated". In the present study, the mean score of this section, in the two intervention groups was significantly lower than that of the control group after education because in the animation and game, the messages were a combination of threat and efficiency. To compare these results, there were no studies examining the fear control process in CL. However, research in other fields revealed that because fear is a distressing feeling, if people do not know the right way to overcome the threat or reduce the threat, they try to eliminate the unpleasant feeling of threat through fear control process [34, 35].

### Danger control process

The main goal of EPPM-based educational interventions is to achieve a danger control process. In this work, to evaluate the danger control process, the variables of intention and behavior were measured and after the education, the average score change in the game group was slightly higher than that in the animation group. Although this difference was not of significance, the game might have a greater impact owing to the exchange of views during the game and creating a lively environment. The results of the present study were similar to those of the EPPM-based studies in other fields, where the use of threat and efficiency messages simultaneously improved the danger control process [34, 36].

High intention is a good predictor of behavior although the gap between intention and behavior should not be overlooked [29]. There may be an intent to behave before education and only the path of behavior should be facilitated, such as informing people of the probable time of bite, how to get insect repellents, and the skills to use it. Of course, when social mechanisms are also involved in the preventive behavior in these circumstances, the determination and use of such mechanisms should be considered. In the present study, an attempt was made to focus on the influencial factors resulting in the intention to take a proper behavior. Nonetheless, certain factors, such as follow-up and telephone calls with relevant organizations for a timely collection of construction debris and garbages, will not be achieved by providing educational materials alone and require the cooperation of relevant agencies [24]. It seems that in addition to having the correct knowledge and perceptions, one solution is to promote cross-sectoral participation. The results obtained in terms of intention and behavior were in accordance with those of other studies in the field of CL prevention [13, 24].

## Conclusion

The results of this study demonstrated that if educational programs contain a combination of threat and efficiency messages, people would enter the danger control process and overcome fear and threat. Providing similar educational content with both game and animation methods showed that both methods had an almost same effect. Furthermore, the cost-effectiveness of the intervention should be always considered; although animation production was more costly, it had the advantage of being easy to use in future periods and for other adolescents.

### The weak and strong points

One of the strengths of this study was production of educational content for the prevention of cutaneous leishmaniasis in the form of animation, which can be used in later periods. In addition, the card game can be useful with distribution in schools. One of the weaknesses and limitations herein was the prevalence of COVID-19 disease and school closures, which made in-person education impossible**.** If there were no COVID-19 restrictions, the students could watch the animation at school and have a group discussion. In the game group, the students would play together, which could have possibly contributed to an increased effectiveness of the educational intervention.

## Supplementary Information

**Additional file 1.** 

## Data Availability

All the data generated or analyzed during this study are included in this published article [and its supplementary information files].

## Abbreviations

EPPM: Extended Parallel Process Model
CL: cutaneous leishmaniasis
